# Molecular Imaging of Brain Tumor-Associated Epilepsy

**DOI:** 10.3390/diagnostics10121049

**Published:** 2020-12-05

**Authors:** Csaba Juhász, Sandeep Mittal

**Affiliations:** 1 Departments of Pediatrics, Neurology, Neurosurgery, Wayne State University School of Medicine, Detroit, MI 48201, USA; 2 PET Center and Translational Imaging Laboratory, Barbara Ann Karmanos Cancer Institute, Detroit, MI 48201, USA; 3 Virginia Tech Carilion School of Medicine, Roanoke, VA 24016, USA; sandeepmittal@vt.edu; 4 Carilion Clinic Neurosurgery, Roanoke, VA 24014, USA; 5 Fralin Biomedical Research Institute, Roanoke, VA 24016, USA

**Keywords:** brain tumors, glioma, epilepsy, seizures, magnetic resonance imaging, positron emission tomography, peritumoral cortex, amino acid, neuroinflammation, glutamate

## Abstract

Epilepsy is a common clinical manifestation and a source of significant morbidity in patients with brain tumors. Neuroimaging has a pivotal role in neuro-oncology practice, including tumor detection, differentiation, grading, treatment guidance, and posttreatment monitoring. In this review, we highlight studies demonstrating that imaging can also provide information about brain tumor-associated epileptogenicity and assist delineation of the peritumoral epileptic cortex to optimize postsurgical seizure outcome. Most studies focused on gliomas and glioneuronal tumors where positron emission tomography (PET) and advanced magnetic resonance imaging (MRI) techniques can detect metabolic and biochemical changes associated with altered amino acid transport and metabolism, neuroinflammation, and neurotransmitter abnormalities in and around epileptogenic tumors. PET imaging of amino acid uptake and metabolism as well as activated microglia can detect interictal or peri-ictal cortical increased uptake (as compared to non-epileptic cortex) associated with tumor-associated epilepsy. Metabolic tumor volumes may predict seizure outcome based on objective treatment response during glioma chemotherapy. Advanced MRI, especially glutamate imaging, can detect neurotransmitter changes around epileptogenic brain tumors. Recently, developed PET radiotracers targeting specific glutamate receptor types may also identify therapeutic targets for pharmacologic seizure control. Further studies with advanced multimodal imaging approaches may facilitate development of precision treatment strategies to control brain tumor-associated epilepsy.

## 1. Introduction

Clinical seizures are among the most common manifestations of brain tumors: approximately 30–50% of patients with neoplastic lesions present with seizures [1]. The incidence of seizures exceeds 90% in patients with low-grade gliomas and glioneuronal tumors [2,3]. Conversely, high-grade brain neoplasms have a lower prevalence of tumor-associated epilepsy. Seizures are a significant source of morbidity in patients with brain tumors, therefore, optimal seizure control, in addition to elimination of the tumor mass, is a major goal of clinical management [4].

Brain tumor-associated epileptogenic regions almost invariably extend beyond the actual magnetic resonance imaging (MRI)-detected lesion. Intraoperative electrocorticography (ECoG) can identify electrographic abnormalities around tumor margins. However, the location and extent of such epileptic regions are highly variable across subjects. Indeed, our studies comparing tumoral lesion margins with electrodes showing early ictal involvement (seizure onset and early spread) on intracranial electroencephalography (EEG) demonstrated that a portion of these electrodes extended at least 1.5 cm beyond the tumor margin in about 90% of the cases [5]. Overall, almost half of the ictally involved electrodes were >1.5 cm from the tumor margin.

Since complete resection of the epileptogenic cortex is associated with a higher likelihood of seizure-free outcome in such tumors [5,6], accurate presurgical delineation of such regions is essential to minimize persistence of seizures postoperatively. Non-invasive detection of such peritumoral epileptogenic regions can help design tailored resections that ensures that not only the tumor mass is resected completely but the surrounding epileptic cortex is also fully removed for post-surgical seizure control while preserving functionally essential cortical areas.

Brain MRI with and without contrast administration is highly accurate and widely used in the clinical practice to identify brain tumors, including both primary brain tumors and metastatic lesions. Structural MRI can visualize the tumor mass and peritumoral edema, but it cannot delineate functional or metabolic abnormalities associated with epileptogenicity. Studies with advanced imaging modalities, including positron emission tomography (PET) and advanced MRI techniques, provided accumulating data, suggesting that neuroimaging has the ability to visualize brain regions specifically associated with brain tumor-induced epileptogenicity. In this paper, we review key studies addressing this issue, mostly focusing on gliomas. We were motivated for this work because previous reviews did not focus on this aspect of brain tumor imaging. We sought to answer the following questions: Can PET imaging delineate the epileptogenic component of the tumor and/or peritumoral brain, and if so, which PET radiotracers hold promise to achieve this? What mechanisms may drive the imaging changes in peritumoral epileptogenic regions? Is tumoral tracer uptake on PET associated with clinical epilepsy characteristics? Can PET variables predict post-treatment seizure outcome? Can advanced imaging of neurotransmitters and their receptors provide useful information regarding tumor-associated epileptogenesis?

## 2. PET Studies in Brain Tumor-Associated Epilepsy

While several PET tracers have been used for imaging various cancer types, including brain tumors, and others are more useful to identify epileptic brain regions, a subset of PET tracers have been used in both fields: they can identify and characterize brain tumors and at the same time were found to be useful to study the epileptic brain, e.g., to identify the epileptic cortex and hippocampus for surgical resection (Table 1).

These PET tracers possess characteristics that can be particularly useful to visualize and measure tracer uptake in epileptogenic lesions, including tumors, as well as identify the peritumoral epileptic cortex.

### 2.1. PET Radiotracers Used in Both Neuro-Oncology and Epilepsy Imaging

Clinically, the most commonly used PET radiotracer is ^18^F-2-fluoro-2-deoxy-D-glucose (FDG) for both tumor and epilepsy imaging. In neuro-oncology, FDG uptake has some ability to differentiate low-grade from high-grade brain tumors, but it is most useful to differentiate tumor progression from radiation-induced brain changes in previously treated gliomas and metastatic brain tumors [7]. High FDG uptake in general is consistent with high-grade tumors, while low-grade gliomas and radiation injury typically show relatively low FDG uptake. In human epilepsy, FDG PET is often used during presurgical evaluation, especially when MRI shows no clear epileptic lesion (i.e., “non-lesional” cases) [8,9]. Epileptic cortex can be identified as areas with focal hypometabolism in the interictal state, while seizures during the tracer uptake period can induce hypermetabolism [10,11]. However, in the interictal state, low FDG uptake on PET is not able to differentiate epileptic vs. non-epileptic tumors or tumor portions, and this method is also not reliable to delineate the margins of peritumoral epileptic cortex.

Unlike FDG, a number of PET radiotracers have been shown to accumulate in both low-grade brain tumors and, in some cases, epileptic non-tumoral lesions and cortex. Most notably, amino acid PET emerged recently as a useful ancillary imaging tool that found a number of clinical applications in neuro-oncology. The most commonly used amino acid PET tracers, listed in Table 2, all utilize the L-type amino acid transporter system (particularly, the large neutral amino acid transporter 1 [LAT1]) to cross the blood-brain barrier and enter the tumor tissue. Once in the tumor, however, these tracers have different metabolic fates (Table 2, also see detailed review in Juhasz et al. [12]), which likely accounts for their differences in tumoral uptake characteristics.

Notably, some of these tracers have no or minimal tumoral metabolism (such as O-(2-^18^F-fluoroethyl)-L-tyrosine [FET]), thus, their accumulation reflects tumoral amino acid transport rates. Others can be metabolized via specific enzymes of certain metabolic pathways, and this can be exploited to image and quantify the activity of these pathways. Interestingly, some of these amino acid tracers have also shown accumulation in epileptogenic brain lesions and even in non-lesional epileptic foci, as discussed below. In addition, PET tracers targeting neuroinflammatory processes, especially those binding to the translocator protein (TSPO, localized predominantly on the outer mitochondria membrane of activated microglia), can accumulate in both gliomas and epileptic tissue [13].

### 2.2. Amino Acid PET in Epileptogenic Tumors and Peritumoral Epileptogenic Cortex

**Figure 1:**
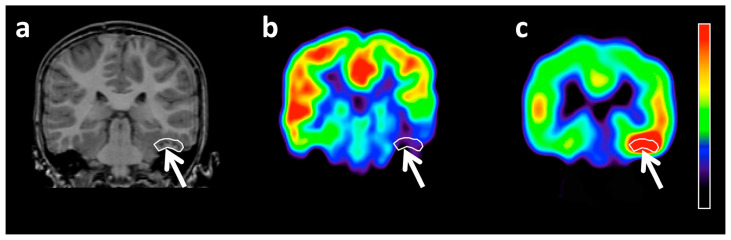
Coronal slices of magnetic resonance imaging (MRI) (**a**), ^18^F-2-fluoro-2-deoxy-D-glucose (FDG) PET (**b**), and α-[^11^C]methyl-L-tryptophan (AMT) PET (**c**) images of a 10-year-old girl with a non-enhancing left inferior temporal dysembryoplastic neuroepithelial tumor (DNT) (arrow). FDG PET showed hypometabolism (FDG uptake tumor/cortex ratio: 0.45), while AMT PET demonstrated markedly increased tryptophan uptake (AMT uptake tumor/cortex ratio: 1.27) in the lesion. The colors on the bar represent a relative scale for the PET images, where the red areas include voxels with the highest and those with deep blue, the lowest radioactivity values within the brain. (Reproduced with permission from Figure 1 of Alkonyi et al. [21]).

Amino acid PET imaging has been endorsed recently by the Response Assessment in Neuro-Oncology working group and the European Association for Neuro-Oncology for clinical use, including differentiating brain tumors from non-tumorous lesions, glioma grading, delineating tumor extent for resection or radiation planning treatment monitoring, as well as detecting progression in gliomas and metastatic brain tumors [7,14,15]. In addition, a number of studies focused on the ability of amino acid PET to detect epileptogenic brain tumors and delineating peritumoral epileptic cortex. Our group published several papers demonstrating that α-[^11^C]methyl-L-tryptophan (AMT), a carbon-11-labeled derivative of the essential amino acid tryptophan, can accumulate selectively in both tumorous and non-tumorous epileptic lesions. The first study to show this involved children with tuberous sclerosis complex, where high AMT uptake was confined to a subset of epileptogenic tubers (but not in non-epileptogenic tubers) as defined by intracranial EEG monitoring [16]. Removal of such high-uptake lesions has led to excellent postsurgical seizure outcome [17]. Subsequent studies demonstrated high AMT uptake in selected epileptic cortical malformations (such as focal cortical dysplasia) [18,19], as well as in low-grade gliomas and neuroepithelial tumors that show almost invariably low uptake on FDG-PET [20,21] (Figure 1). Detailed comparisons to scalp and intracranial EEG data showed up to 100% lobar specificity for increased focal AMT uptake to detect the epileptic focus in patients with tuberous sclerosis and cortical developmental malformations [17,18]. These studies suggested that high AMT uptake in non-tumorous lesions and low-grade, hypometabolic tumors may be an imaging marker of epileptogenicity.

Accumulation of AMT in these lesions is likely related to, at least partly, conversion of AMT to α-[^11^C]methyl-L-kynurenine via indoleamine-2,3,-dioxygenase 1 (IDO1). IDO1 is the initial and rate-limiting enzyme of the inflammatory and immunosuppressive inflammatory kynurenine pathway, and upregulation of this enzyme (along with tryptophan 2,3-dioxygenase 2 [TDO2]) has been reported in various tumors to promote tumoral immune resistance [22,23]. Our studies in various gliomas, glioneuronal tumors, and meningiomas demonstrated that high tumoral IDO1 expression on immunohistochemistry (and, in the case of meningiomas, high TDO2 expression) was associated with altered AMT metabolic rates estimated by tracer kinetic analysis from dynamic PET scanning [24,25]. Using imaging-guided biopsy samples, we have also showed the overexpression of IDO1, along with other inflammatory markers (such as interleukin β and its receptor) in an AMT-avid inflammatory lesion (mimicking a tumorous lesion) associated with new-onset status epilepticus [26]. This study provided additional proof-of-concept data on the ability of AMT-PET to detect inflammatory changes in epileptogenic lesions.

**Figure 2:**
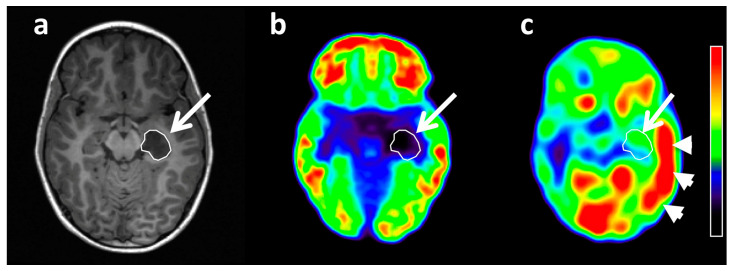
Co-registered axial MRI (**a**), FDG PET (**b**), and AMT PET (**c**) images of a 6-year-old boy with a left medial temporal DNT (arrow). FDG PET demonstrated severe glucose hypometabolism of the tumor (FDG uptake tumor/contralateral cortex ratio: 0.29), and AMT PET showed no prominent tryptophan accumulation in the tumor volume (AMT uptake ratio: 0.99). However, an extensive area of ipsilateral cortex (mostly temporal) showed a marked increase of AMT uptake (arrowheads) (ipsilateral/contralateral cortex ratio: 1.21). Note that tracer accumulation seen in the bilateral occipital cortex and in the visualized portions of the basal ganglia, seen on the AMT PET image, is likely physiologic, because these regions (along with the cerebellum) are among the highest uptake regions in the brain in both healthy controls and epilepsy patients (regardless of the location of the presumed epileptic focus) [28]. The colors on the bar represent a relative scale for the PET images, where the red areas include voxels with the highest and those with deep blue the lowest radioactivity values within the brain. (Reproduced with permission from Figure 2 of Alkonyi et al. [21]).

Dysembryoplastic neuroepithelial tumors (DNTs) are highly epileptogenic lesions that often manifest with early onset focal seizures. Cortex surrounding or overlying these lesions are often epileptic and may encompass focal cortical dysplasia; therefore, ECoG mapping of these peritumoral regions can optimize resection margins and surgical outcome [27]. In our multimodal imaging study of 11 patients with DNT [21], high AMT uptake was found not only in most DNTs but also in peritumoral cortex in 2 cases in the ipsilateral temporal or parietal cortex (Figure 2). These extratumoral cortical regions with high AMT uptake showed no structural abnormality on MRI and no glucose hypometabolism on FDG PET (Figure 2), but they showed interictal spiking on intraoperative ECoG. The cortical regions were not fully included in the resection, and both patients continued to have seizures. These findings supported the notion that high peritumoral cortical AMT uptake in low-grade tumors may be also an imaging marker of epileptogenicity.

Although published data are less abundant on the role of imaging epileptic cortex in brain tumors with other (non-AMT) amino acid PET tracers, a few studies provided intriguing positive findings in this regard. For example, increased [^11^C]-methionine (MET) uptake was reported in the cortex adjacent to a recurrent metastatic lesion associated with focal epilepsy with motor seizures [29]. The high uptake area included the sensorimotor cortex, and removal of the lesion has led to cessation of the seizures. It remained unclear how much of the MET-positive cortex was included in the resection, but the authors discussed the probability that the high-uptake area was associated with epileptogenicity.

**Figure 3:**
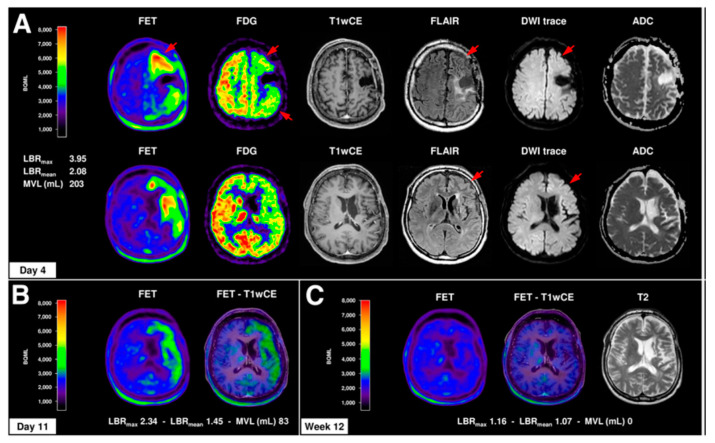
Cortical amino acid uptake in O-(2-^18^F-fluoroethyl)-L-tyrosine (FET) PET in the course of a prolonged postictal episode. A 44-year-old man with clinically stable anaplastic astrocytoma WHO III without any evidence of a residual tumor over years presented with tonic-clonic seizures followed by severe and prolonged postictal symptoms (global aphasia, right-sided hemiplegia, and hemineglect) over an 8-week period. (**A**) MRI (day 1) and FET PET (day 4) showed distinct increased and extended cortical FET uptake of the left hemisphere (LBR_max_, 3.95; LBR_mean_, 2.08) with frontal and temporal accentuation, corresponding to slight cortical vasogenic and cytotoxic edema (T2/FLAIR, DWI/ADC) without contrast enhancement (T1wCE). EEG monitoring, ^18^F-FDG PET (glucose hypometabolism, red arrows), and 99mTc-HMPAO SPECT (showing hypoperfusion), however, revealed no evidence of status epilepticus. (**B**) For FET PET 11 days after symptom onset and 7 days after the first FET PET, slight regression of cortical FET uptake (LBR_max_, 2.34; LBR_mean_, 1.45) was observed. (**C**) The patient slowly recovered within 8 weeks after seizure onset. FET PET and MRI 12 weeks after symptom onset demonstrated complete recovery of cortical FET uptake and brain edema; only residual cortical atrophy in T1 and T2/FLAIR sequences remained. (Reproduced with permission from Figure 3 of Hutterer et al. [30]). LBR: lesion-to-brain ratio.

Another study, using FET PET imaging, provided compelling data indicating peri-ictal, transient increase of cortical amino acid transport in 10 patients with an epileptogenic brain lesion (including 8 WHO grade II-IV gliomas, most of them previously treated with or without residual tumor) [30]. In seven of these patients, FET PET/CT was done during a period of serial seizures, status epilepticus, or prolonged postictal symptoms, and the scans showed increased FET uptake strictly following the cortical ribbon of seizure-affected brain areas in the regions corresponding to structural MRI changes (Figure 3). FET accumulation was seen in areas both with and without contrast enhancement on co-registered MRI. Four patients with serial seizures showed widespread cortical FET accumulation spreading into 2 or 3 lobes, combined with cortical vasogenic and cytotoxic edema and partial contrast enhancement in MRI; these patients showed prolonged postictal symptoms lasting 1–6 weeks. PET scans performed weeks before, or as a follow-up weeks after the peri-ictal scans, showed normal (low) FET uptake in the same brain regions (Figure 3).

These data suggested reversible, seizure-induced cortical increases of amino acid uptake in this cohort. The authors also performed histopathologic evaluation and immunostaining of LAT1, LAT2, and CD98 protein expression in selected biopsy specimens to study the potential role of high transporter expression in the observed high FET uptake on PET. Neuropathologic evaluation of seizure-affected brain yielded cortical brain edema with reactive astrocytosis and microglial activation without evidence of tumor cell infiltration. In the seizure-affected cortex, LAT1/LAT2/CD98 amino acid transporter showed strong expression in neurons and brain endothelial cells and, to a lesser degree, in reactive astrocytes, especially adjacent to brain capillaries. In the glioma-infiltrated cortex, neurons also showed a pronounced expression of LAT1/LAT2/CD98, in particular where glioma cells were adjacent to neurons [30]. Overall, these results indicated a seizure-induced increase of cerebral amino acid transport in peritumoral cortex that appears to be mediated by neuronal, endothelial, and to a lesser degree, astroglial LAT1/LAT2/CD98 expression. The authors noted that such peri-ictal, transient increases on PET may be falsely interpreted as tumor progression.

### 2.3. Imaging Activated Microglia in Epileptic Foci, Gliomas, and Peritumoral Cortex

**Figure 4:**
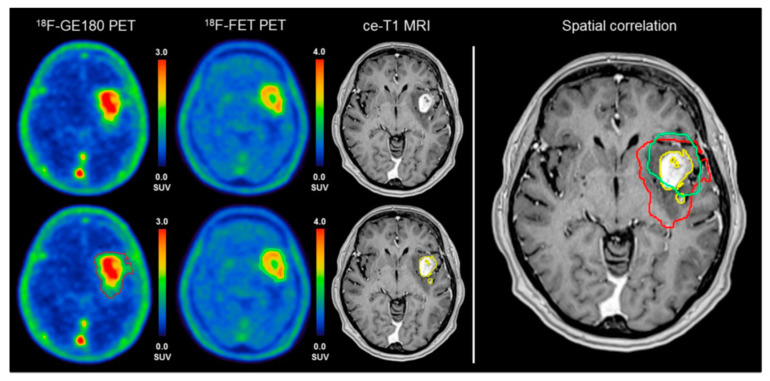
In a newly diagnosed, isocitrate dehydrogenase (IDH)-wildtype glioblastoma, a moderate spatial overlap was found between the biological tumor volume from TSPO (GE-180) PET (red line) and amino acid (FET) PET (green line). Note that both PET volumes extended way beyond the boundaries of the contrast-enhancing volume (ce-T1), and the high TSPO volume further extended behind the FET PET volume. (Reproduced with permission from Figure 1 of Unterrainer et al. [36]).

In the past decade, there has been increasing evidence that TSPO PET is able to detect various epileptogenic lesions that contain an increased density of activated microglia. While earlier reports demonstrated high TSPO binding in patients with severe and/or prolonged epileptic seizures [31], more recent studies also detected increased TSPO binding in the interictal state both in epileptic hippocampi and in neocortical epileptic foci and focal cortical dysplasia [32,33,34]. PET studies combining TSPO and amino acid tracers in both preclinical models and in patients with cerebral gliomas demonstrated that the areas with increased accumulation overlap only partially: high TSPO binding can extend beyond the contrast-enhancing tumor mass and even substantially beyond areas with high amino acid uptake in peritumoral areas [35,36] (Figure 4).

Considering the above PET findings detecting high TSPO binding in epileptic foci and lesions, one can postulate that such TSPO-positive peritumoral regions may coincide with tumor-associated epileptic foci. This hypothesis is supported by recent preclinical data showing widespread and persistent microglial infiltration extending beyond the tumor mass as an early process during the period of epileptogenesis [37]. These cortical microglia infiltrations are associated with recurrent spreading depolarization during this period and, along with other neuroinflammatory processes, may contribute to recurrent clinical seizures [38,39]. However, direct comparison with areas of microglia infiltration (detected by TSPO imaging and/or histopathology) and cortical electrophysiologic data have yet to be reported in human epileptogenic gliomas.

### 2.4. Associations between PET Imaging, Clinical Seizure Variables, and Seizure Outcome

A few studies demonstrated the ability of amino acid PET parameters to predict clinical seizure variables and outcome in terms of seizure control. The first study showing such a correlation involved glioneuronal tumors [40]. In this study of 36 patients, FET uptake, measured in the MRI-defined lesions, showed a wide range of tumor/normal standardized uptake value (SUV) ratios (range: 1–5; median: 1.3). These values were not able to differentiate the actual tumor type (DNT vs. ganglioglioma; available in 22 cases with histopathology data). All patients had refractory epilepsy, but seizure frequency was not associated with the level of FET uptake. In contrast, there was an inverse correlation between SUVs (and SUV ratios) and age at seizure onset (r = −0.51, *p* < 0.01 for the latter in Pearson’s correlation): i.e., patients with FET-enhancing lesions were significantly younger at epilepsy onset than those with low FET uptake (14 vs. 27 years, respectively). These data indicate a substantial heterogeneity of amino acid transport in grade I glioneuronal tumors, probably reflecting differences in the activity of LAT1 transporter. Why tumors with high uptake manifest earlier with clinical seizures remains to be elucidated.

In a subsequent study of 33 patients with low-grade gliomas, serial MRI and amino acid PET scans (n = 125 in total, using FET or MET tracers) were used to evaluate response to chemotherapy (temozolomide) [41]. Twenty-five patients showed clear metabolic responses in the form of an exponential drop of the active tumor volume measured on PET (25% decrease in a mean 2.3 months follow-up period). MRI tumor volume decreases lagged behind the PET responses with slower, linear changes. Interestingly, the interval change in metabolic tumor volumes during therapy also predicted how well the seizures became controlled during this period. Among the 17 participants under stable antiepileptic drug doses, chemotherapy reduced the active tumor volumes by 22% ± 27% in participants with no/limited seizure control (0–50% seizure frequency reduction) and by 64% ± 28% in those with good seizure control (>50% frequency reduction, including seizure freedom). In contrast, tumor volume changes on T2-weighted MRI images were not associated with seizure frequency reductions. The authors speculated that improved seizure control may not rely exclusively on reduced compression of neuronal structures by the tumor but also on the reduction of magnitude and spatial extent of local metabolic disequilibrium. They also postulated that mechanisms driving improved seizure control may involve the glutamatergic system. Gliomas release glutamate, an excitatory neurotransmitter, and activate neuronal glutamate receptors [42]. FET uptake can increase during periods of frequent seizures in the peritumoral cortex and resolve after cessation of epileptic activity, as discussed above [30]. Whether transport of both types of amino acids, which use different transport systems under physiological conditions, is somehow related in gliomas, remains to be evaluated. The role of glutamate in glioma-associated seizures measured in tumor and peritumoral brain tissue can be directly addressed by glutamate MR imaging, as discussed in the next section.

## 3. Imaging Neurotransmitters and Their Receptors in Epileptogenic Brain Tumors

While the PET studies discussed above addressed mostly tumoral and peritumoral amino acid transport and neuroinflammatory changes, the mechanisms of tumor-associated epileptogenicity are clearly multifactorial and may involve both pathologic glutamate release and gamma-aminobutyric acid (GABA)-mediated disinhibition of surrounding neurons [43,44]; therefore, an overview of molecular imaging of glioma-related epilepsy would not be complete without discussing imaging options of these neurotransmitters and their receptors.

### 3.1. Imaging Glutamate Levels and Glutamate Receptors

The excitatory amino acid neurotransmitter glutamate accumulates in peritumoral tissue as it is released via the “system x_c_” (SXC) cystine-glutamate anti-porter [45], and glutamate concentrations can exceed 100 μM in periglioma brain [46]. Expression levels of SXC in glioma patients correlated with the severity of tumor-associated seizures [47].

Glutamate levels in the tumor mass and peritumoral brain tissue can be assessed non-invasively by proton magnetic resonance spectroscopy (^1^H-MRS), utilizing single voxel spectroscopy [48] or chemical shift imaging [49]; or, more recently, by the chemical exchange saturation (CEST) technique [50]. The CEST technique measures proton exchange between the exchangeable protons of the solute with the pool of bulk water protons [51]. When the magnetization from the exchangeable protons is saturated with a frequency-selective radiofrequency pulse, there is a proportional decrease of the water signal due to accumulation of saturated protons in the water pool. The signal difference obtained with and without saturation of the solute pool is measured as the CEST effect. The amine proton in the glutamate molecule resonates at 3 parts per million (ppm) down field from water, and the exchange rate is within a slow to intermediate exchange regime, making glutamate an ideal neurotransmitter for CEST imaging with high magnetic field (≥7T) MRI scanners [51,52]. GluCEST has at least two magnitudes higher sensitivity than traditional ^1^H MRS for measuring glutamate levels, and this method images glutamate in vivo at higher spatial resolution than can be achieved with single-voxel MRS or CSI [51,52].

**Figure 5:**
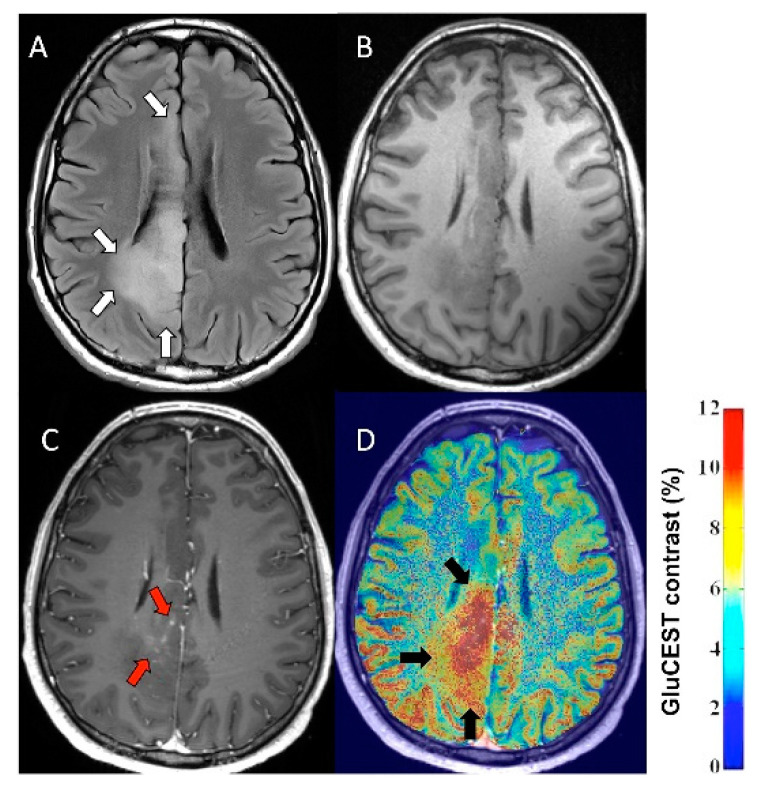
GluCEST contrast vs. gadolinium enhancement in a WHO grade II oligodendroglioma. (**A**) 3T FLAIR images in the axial GluCEST acquisition plane, tumor identified by white arrows. (**B**) 3T T1-weighted images without gadolinium contrast in the axial GluCEST acquisition plane. (**C**) 3T T1-weighted images with gadolinium contrast in the axial GluCEST acquisition plane with evidence of nodular, wispy mesial contrast enhancement (red arrows). (**D**) 3T T1-weighted images with gadolinium coregistered with GluCEST contrast maps. Increased GluCEST contrast in a region overlapping but extending beyond the area of gadolinium enhancement (black arrows). (Reproduced from Figure 2 of Neal et al. [54]).

In a proof-of-concept study, Davis et al. [53] demonstrated the ability of GluCEST MRI to lateralize the epileptic focus in 4 patients with non-lesional temporal lobe epilepsy. In this small pilot study, the epileptogenic hippocampus showed increased GluCEST contrast as compared to the contralateral hippocampal values. More recently, Neal et al. [54] applied this technique in 9 patients with WHO grade II-III supratentorial diffuse gliomas, including 7 with a history of seizures. The authors analyzed glutamate levels in both tumoral and peritumoral regions, separately. Tumor GluCEST contrast was higher in patients with oligodendroglial tumors compared to those with an astrocytic histology (*p* = 0.048), while peritumoral GluCEST contrast did not differ based on histology. Increased GluCEST contrast often extended beyond the contrast-enhancing tumor portions (Figure 5).

Peritumoral GluCEST contrast was significantly higher in patients who had at least one seizure in the month prior to 7T MRI as compared to those who were seizure free in the prior month (*p* = 0.038) and the contrast was also higher in those with drug resistant epilepsy (*p* = 0.029). The authors acknowledged the need for further validation of this method, but they noted that GluCEST offers a potential means to identify patients at high risk for seizures; this approach may also help select those who likely benefit from drugs targeting glutamate, such as the AMPA receptor blocker perampanel, a clinically used antiseizure drug that may be effective to prevent glioma-associated seizures [55,56]. The authors of this study also discussed potential limitations of the GluCEST method, including the fact that GluCEST contrast is sensitive to changes in pH, with an inverse relationship with CEST signal at 3 ppm [52,57]. This could pose a challenge to estimate glutamate within the glioma mass where extra-cellular pH is often acidic, while intracellular pH is typically alkaline, and low pH can favor tumor growth and invasion [58,59]. The non-significant correlation between GluCEST and MRS glutamate in the tumor mass, found by Neal et al. [54], may reflect both the incomplete overlap of the MRS and GluCEST voxel and the potential contribution of altered pH to the GluCEST signal. This latter issue may be less relevant in the peritumoral cortex where the two glutamate signals correlated well in the same study.

Despite the central importance of glutamate neurotransmission in brain physiology and in neurological disorders including epilepsy, specific PET ligands for receptors of glutamate have been scarce, although recent efforts have made progress in this respect (reviewed by Kim et al. [60]). For example, a recent study reported promising findings for AMPA receptor imaging of human epilepsy [61]. In addition, successful detection of abnormal metabotropic glutamate receptor type 5 has been reported in the epileptic hippocampus [62]. While there are still relatively limited selective and sensitive approaches for molecular imaging of ionotropic and metabotropic glutamate receptors, the emerging new ligands can provide new tools, alone or combined with glutamate MRI, to assess peritumoral epileptogenicity associated with abnormal glutamate release and neurotransmission.

### 3.2. Imaging Tissue GABA Levels and GABA_A_ Receptors

Peritumoral hyperexcitability is not exclusively due to elevated glutamate levels, and the role and mechanism of impaired inhibition by GABA in this process has been also established [63]. GABA_A_ receptors are ligand-gated ion channels whose response to GABA is largely dictated by the chloride ions (Cl^−^) in post-synaptic neurons, which is primarily established by the activity of the K^+^-Cl^−^ cotransporter KCC2. Studies with preclinical glioma models demonstrated that increased concentrations of glutamate can lead to downregulation of KCC2, leading to GABAergic disinhibition in the peritumoral cortex; this mechanism may contribute to glioma-associated epileptogenesis in humans [63,64].

There have been only a few studies attempting the measurement of GABA concentrations in human epileptic foci using ^1^H-MRS: the findings were not robust in temporal lobe epilepsy [65], while increased GABA concentrations were reported in tubers of 4 children with tuberous sclerosis [66]. The authors interpreted these increases as likely compensatory due to reduced GABA receptor function (supported by decreased ^123^I-iomazenil binding on SPECT imaging in the same patients). A more recent study used proton high-resolution magic angle spinning spectroscopy to measure multiple metabolites in gliomas and found both GABA and glutamate concentrations to be reduced in isocitrate dehydrogenase (IDH)-mutated lesions; however, these data were not studied from the prospect of epileptogenicity [67].

GABA_A_ receptors can be readily evaluated in the living brain by measuring the binding of the GABA_A_-receptor antagonist ^11^C-flumazenil. This approach has been used extensively in evaluating both temporal and extratemporal epileptic foci and lesions, which often show decreased binding on ^11^C-flumazenil PET, including in the non-lesional epileptic cortex, as compared to data from intracranial EEG [68,69,70]. The potential utility of ^11^C-flumazenil PET in brain tumor imaging has been first tested in 5 patients with epileptogenic DNTs [71]. Decreased ^11^C-flumazenil binding was detected in all 5 tumors, and additional extratumoral decreases were also found in the peritumoral cortex and ipsilateral mesial temporal regions. In a subsequent study, decreased ^11^C-flumazenil binding was also reported in peritumoral regions in patients with low-grade brain tumors (including gliomas, a DNT and a meningioma) [72]. While promising, no further studies applying rigorous correlations with intracranial EEG data were pursued to establish the relation between such areas of low GABA_A_ receptor binding and peritumoral epileptogenicity. Imaging with ^11^C-flumazenil did not gain widespread clinical application, partly because of its short half-life of carbon-11 (20 min); also, the usual quantification methods of tracer binding could not differentiate changes due to receptor density vs. affinity. Recent studies suggested that a F-18-labeled flumazenil analog (with a 110-min half-life) has similar imaging characteristics and is amenable to more widespread clinical application (reviewed by Hodolic et al. [73]); however, this tracer is available only in a few centers, and its potential utility in brain tumor-associated epilepsy is yet to be explored.

## 4. Conclusions

Although the primary goal of brain tumor imaging is detection, delineation, and characterization of the tumor itself, recent imaging studies provided increasing evidence about their ability to provide information related to tumor epileptogenicity. Most of these studies focused on gliomas and glioneuronal tumors that often manifest with seizures. The review of these studies provided some answers to the five questions posed in the Introduction, as summarized below:

1. Can PET imaging delineate the epileptogenic component of the tumor and/or peritumoral brain, and if so, which PET radiotracers hold promise to achieve this? PET imaging with some amino acid radiotracers can detect increased uptake not only in the tumor mass but also in the peritumoral epileptic cortex both in the interictal and peri-ictal state. Data from PET imaging of AMT, a tryptophan derivative radiotracer, indicate that increased cortical AMT uptake is a strong imaging marker of epileptogenicity. In addition, accumulation of PET ligands binding to TSPO on activated microglia has been reported in tumor-associated epilepsy.
2. What mechanisms drive the imaging changes in peritumoral epileptogenic regions? Increased amino acid uptake on PET imaging is likely due to overexpression of the LAT1 amino acid transporter. Increased AMT uptake may also indicate activation of the neuroinflammatory kynurenine pathway. Peritumoral increase of TSPO PET tracers is driven by activated microglia and may involve epileptic regions as suggested by preclinical tumor models; however, the electrophysiology correlates of these PET abnormalities in humans need to be established before TSPO PET could be used to guide tailored resections of peritumoral epileptic cortex.
3. Is tumoral tracer uptake on PET associated with clinical epilepsy characteristics? While such data have been scarce, in one study with glioneuronal tumors, FET uptake in the tumors did not correlate with seizure frequency but showed an inverse correlation with the age at seizure onset: patients with FET-enhancing lesions were significantly younger at epilepsy onset than those with low FET uptake. Why tumors with high amino acid uptake manifest earlier with clinical seizures remains to be elucidated.
4. Can PET variables predict post-treatment seizure outcome? Limited studies with amino acid PET showed promise for metabolic tumor volumes to detect early treatment response before structural MRI changes and predict seizure outcome during glioma chemotherapy.
5. Can advanced imaging of neurotransmitters and their receptors provide useful information regarding tumor-associated epileptogenesis? Advanced MRI, such as glutamate imaging, can detect neurotransmitter changes around epileptogenic brain tumors, although the high-resolution GluCEST technique requires 7T scanners that are currently not widely available. PET imaging of GABA_A_ and specific glutamate receptors may identify receptor abnormalities that can serve as therapeutic targets for personalized pharmacologic seizure control.

Future studies may combine advanced imaging techniques to evaluate metabolic and neuroinflammatory changes, biochemical properties, as well as specific neurotransmitter and receptor abnormalities in the peritumoral cortex of individual patients. Comparisons of multimodal imaging findings to histopathologic and electrophysiologic changes can facilitate development of precision treatment strategies to control brain tumor-associated epilepsy.

## Figures and Tables

**Table 1:** Positron emission tomography radiotracers utilized for both epilepsy and neuro-oncology imaging.

Imaging Target	PET Radiotracers
Glucose metabolic rate	FDG
Amino acid transport/metabolism	AMT
	MET
TSPO on activated microglia	[^11^C]PK11195, [^11^C]PBR28, [^18^F]GE180

FDG: ^18^F-2-fluoro-2-deoxy-D-glucose; AMT: α-[^11^C]methyl-L-tryptophan; MET: [^11^C]-methionine; TSPO: translocator protein.

**Table 2:** Transport mechanism and metabolic process(es) affecting tumoral uptake of the most common amino acid PET tracers.

PET Tracer	Transport	Cellular Metabolism
MET	System L (LAT1)	Transmethylation
		Protein incorporation
FET	System L (LAT1)	None
FDOPA	System L (LAT1)	Aromatic amino acid decarboxylase (to dopamine)
AMT	System L (LAT1)	Tryptophan-hydroxylase (to serotonin)
		Indoleamine 2,3-dioxygenase (to kynurenine)

MET: [^11^C]-methionine; FET: O-(2-^18^F-fluoroethyl)-L-tyrosine; FDOPA: ^18^F-fluoro-dihydroxy-phenylalanine; AMT: α-[^11^C]methyl-L-tryptophan; LAT1: large neutral amino acid transporter 1.
